# Patterns, severity, and outcomes of traumatic brain injury in a regional referral hospital in Kenya: a retrospective cohort study

**DOI:** 10.1097/MS9.0000000000004260

**Published:** 2025-11-04

**Authors:** Ben Ojakapeli, Barasa Juliet Nafula, Laurel Seltzer

**Affiliations:** a School of Medicine, Moi University, Kenya; b School of Medicine, University of Nairobi, Kenya; c School of Medicine, Tulane University School of Medicine, USA

**Keywords:** cohort study, injury outcomes, Kenya, neurosurgery, neurosurgical access, traumatic brain injury

## Abstract

**Background:**

Traumatic brain injury (TBI) is a growing public health burden globally, especially in low- and middle-income countries like Kenya. Inadequate prehospital care and limited health care infrastructure in rural areas hinder optimal TBI management. This study aimed to describe the patterns, severity, and short-term outcomes of TBI at a regional referral hospital in western Kenya.

**Methods:**

A retrospective cohort study was conducted, reviewing the medical records of 738 TBI patients admitted between January 2021 and December 2023. Data were extracted on demographics, injury mechanisms, Glasgow Coma Scale (GCS) scores, imaging, interventions, and discharge outcomes. Descriptive statistics were used to summarize patient characteristic. Chi-square tests and binary logistic regression were used to identify associations and predictors of mortality.

**Results:**

Most patients were males (89%) with a mean age of 30 years. road traffic accidents (RTAs) were the leading cause of injury (61%), followed by assaults (27%) – a figure higher than national and global averages. Mild TBI was most prevalent cases (51.6%). Imaging and neurosurgical intervention rates were low at 34.2% and 2.8%, respectively. At discharge, 59.9% of patients had fully recovered, while the mortality rate was 11.7%. Predictors of mortality included moderate (OR = 8.07) and severe (OR = 61.18) GCS scores and injury from falls (OR = 3.40).

**Conclusion:**

The study highlights the high burden of preventable TBI from RTAs and assaults in this setting. The high assault rate, low imaging access, and limited neurosurgical intervention capacity are major concerns. While the findings are limited to admitted patients and short-term outcomes, they underscore the urgent need for targeted road safety initiatives, improved access to specialized care, and a better understanding of the high prevalence of assault-related TBI to inform policy in similar low-resource settings.

## Introduction

Traumatic brain injury (TBI) poses a significant public health concern worldwide, with about 69 million people worldwide sustaining a TBI each year^[1]^. TBI occurs when external force disrupts brain function and/structure, and can lead to physical, cognitive, behavioral, and emotional impairments, significantly impacting the quality of life of affected individuals^[2]^. In low- and middle-income countries (LMICs) like Kenya, the burden of TBI is on the rise, driven by road traffic accidents, falls, and assaults. Despite the considerable impact of TBI, it is often underreported and poorly addressed in these settings.

Globally, TBI incidence varies, with Africa showing an incidence rate of 801 per 100 000 people, while North America reports a higher rate of 1299 per 100 000 people^[1]^. The incidence of TBI in Kenya is notably high, with road traffic accidents (RTAs) accounting for over half of all cases^[3]^. Young males are disproportionately affected, often in the socially and economically productive age group^[3,4]^. TBI severity, as classified by the Glasgow Coma Scale (GCS), is a critical predictor of patient outcomes, with severe TBI being associated with high mortality and long-term disability^[5]^. However, factors such as delayed treatment, limited access to timely imaging, and the scarcity of specialized neurosurgical care continue to contribute to poor outcomes^[3,4,6]^.

This study was conducted at a regional referral hospital in western Kenya, which serves a large, predominantly rural population. The facility, despite its critical role, faces challenges including a high patient load and resource constraints. To inform clinical management and resource allocation, this study addresses the following research question: what are the patterns, severity, and short-term outcomes of TBI among patients admitted to a regional referral hospital in western Kenya? The findings will contribute valuable data from a high-burden, low-resource setting, offering insights to guide evidence-based interventions and policy development.

## Methods

This was a retrospective cohort study conducted at a regional referral hospital in western Kenya. The study population included all patients with a TBI diagnosis who were admitted to the facility between January 2021 and December 2023. A census of all eligible TBI cases was conducted from the hospital’s admission and discharge records. The study was reported in accordance with the strengthening the reporting of cohort, cross-sectional and case-control studies in surgery (STROCSS) 2025 guidelines^[7]^.

Data were extracted from patient records using a structured, pre-validated data collection tool. The tool captured socio-demographic information, injury mechanism and etiology, GCS score at admission, associated clinical features, imaging performed, and discharge outcomes.

Following data collection, entries were cleaned and organized using Microsoft Excel, then exported to SPSS version 27 for statistical analysis. Descriptive statistics – including frequencies, percentages, means, and standard deviations – were used to summarize patient characteristics, injury patterns, and outcomes. Chi-square tests were conducted to explore associations between categorical variables. Binary logistic regression models were used to identify predictors of mortality. To address the potential for Type 1 error inflation from multiple comparisons, a Bonferroni correction was applied for all chi-square tests, with the significance level adjusted to *P* < 0.05/number of tests. Statistical significance was determined at a 95% confidence level.

Ethical approval for this study was obtained from the Research Unit of the participating hospital and from the National Commission for Science, Technology and Innovation (NACOSTI) in Kenya (NACOSTI/P/25/415 417). Given the retrospective nature of the study, which utilized anonymized patient records, the requirement for individual informed consent was waived.

## RESULTS

A total of 738 patient records were reviewed between January 2021 and December 2023.

### Patient demographics and socio-economic factors

**Figure 1.**
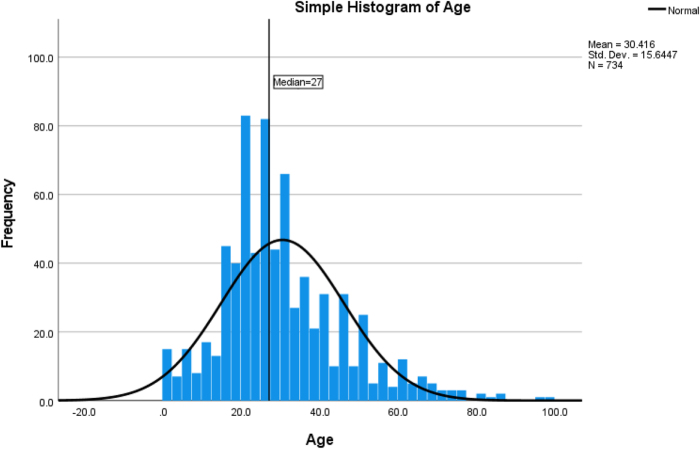
Age distribution for participant patients with traumatic brain injury in a regional referral hospital.

The majority of patients were male (89%, *n* = 658), with a mean age of 30 years and a median of 27 years. (Fig. 1)

Table 1 presents the socio-demographic characteristic of the cohort. A significant proportion of patients did not have a recorded education level (45.9%, *n* = 341) or health insurance (28.5%, *n* = 212). The low rate of documented insurance coverage (6%, *n* = 44) is noteworthy.

**HIGHLIGHTS**

- **Background**: Traumatic brain injury (TBI) is a major public health issue globally and is increasingly prevalent in low- and middle-income countries such as Kenya, largely due to road traffic accidents, falls, and assaults. There is limited data on TBI patterns and outcomes in rural Kenyan referral hospitals.
- **Key findings**:
- Young adult males under 40 years constituted the majority of TBI patients, with road traffic accidents accounting for 61% of cases.
- Only 6% of patients had health insurance, and just 34% underwent imaging despite a large proportion having moderate or severe TBI.
- Severe TBI and falls were independently associated with significantly higher mortality rates.
- Neurosurgical intervention was rare (2.8%), often due to limited specialist availability and critical care infrastructure.
- **Clinical relevance**: The findings underscore the urgent need to strengthen trauma care systems in rural Kenya, particularly through investments in critical care capacity, neurosurgical services, and prehospital care. Public health policies focusing on road safety and alcohol regulation may significantly reduce the burden of TBI.

**Table 1:** Socio-demographic characteristics of patients with traumatic brain injury in a regional referral hospital

Variables (*N* = 738)	Frequency (*n*)	Percentage (%)
Level of education		
None	25	3.4
Primary	219	29.8
Secondary	116	15.8
Tertiary	37	5.0
Unknown	341	46.0
Residence		
Rural	393	53.4
Unknown	99	13.2
Urban	246	33.4
Health insurance status		
Has medical insurance	44	6.0
No medical insurance	482	65.5
Unknown	212	28.5

### Mechanisms, severity, and clinical findings

RTAs were the leading cause of TBI, accounting for 61% (*n* = 450) of all cases. This was followed by assaults (27%, *n* = 196) and falls (7%, *n* = 50). Alcohol consumption at the time of injury was significantly associated with the mechanism of injury (*P* = 0.016). Among those with known alcohol use, the highest percentage of use was reported in assault cases (16.8%; Table 2).

**Table 2:** Association between alcohol use and mechanisms of injury

Total participants (*N* = 738)		Mechanism of injury					*P*-value
		Assault (*N* = 196, 27%)	Fall (*N* = 50, 7%)	Other (*N*= 17, 2%)	Road traffic accident (*N* = 450, 61%)	Work-related injury (*N* = 25, 3%)	
Alcohol use at the time of injury	No	94 (48.0)	40 (80.0)	9 (52.9)	251 (55.8)	17 (68.0)	**0.016**
	Unknown	69 (35.2)	6 (12.0)	6 (35.3)	136 (30.2)	5 (20.0)	
	Yes	33 (16.8)	4 (8.0)	2 (11.8)	63 (14.0)	3 (12.0)	

Regarding injury severity, mild TBI was the most prevalent (51.6%, *n* = 381), with moderate and severe TBI comprising 27.6% (*n* = 204) and 11.9% (*n* = 88) of cases, respectively. Soft tissue injuries were the most common associated injury (61.5%, *n* = 454). Only 2.8% (*n* = 21) of patients received neurosurgical interventions (Table 3).

**Table 3:** Initial Glasgow Coma Score, associated injuries, and neurosurgical intervention for patients with traumatic brain injury

Variables (*N* = 738)	Frequency (*n*)	Percentage (%)
Initial Glasgow Coma Score categorization		
Severe	88	11.9
Moderate	204	27.6
Mild	381	51.6
Unknown	65	8.9
Other associated injuries		
Fractures	109	14.8
Internal organ injury	12	1.6
Soft tissue injury	454	61.5
Finger dislocation	1	0.1
Spinal cord injury	1	0.1
None	161	21.9
Neurosurgical intervention		
No	717	97.2
Yes	21	2.8

Imaging was performed on 34.2% (*n* = 252) of patients, predominantly using CT scans (28.0%, *n* = 207). There was a significant association between injury severity and whether imaging was performed (*P* = 0.004). However, imaging was not performed on majority of patients in the mild (70.4%, *n* = 314) and severe (69.6%, *n* = 39) TBI categories (Table 4; Table 5).

**Table 4:** Imaging procedures performed and their distribution among patients with traumatic brain injury

Imaging done (*n* = 738)	Frequency (*n*)	Percentage (%)
Yes	252	34.2
No	486	65.8
Type of imaging done		
CT	207	28.0
MRI	11	1.5
None	486	65.9
X-ray	34	4.6

**Table 5:** Relationship between whether imaging was done and severity of injury based on Glasgow Coma Scale score

GCS Score rating	No	Yes	*P* = 0.004
Mild	314 (70.4)	132 (29.6)	
Moderate	117 (57.3)	87 (42.7)	
Severe	39 (69.6)	17 (30.4)	

### Patient outcomes

**Figure 2.**
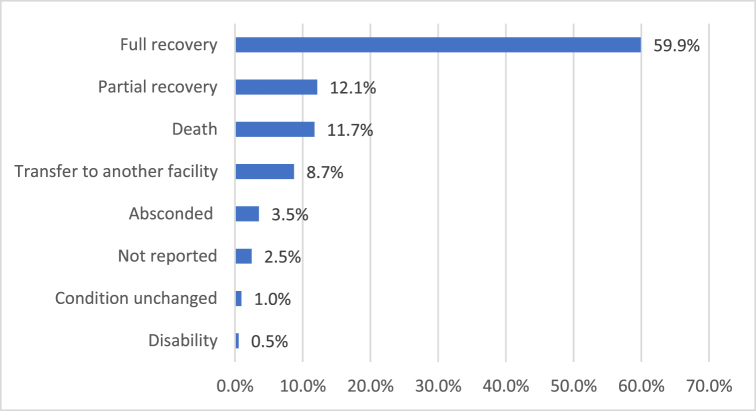
Outcome of patients with traumatic brain injury.

At discharge, 59.9% (*n* = 442) of patients had a full recovery, while 11.7% (*n* = 86) died. Other outcomes included partial recovery (12.1%, *n* = 89) and transfer to another facility (8.7%, *n* = 64; Fig. 2).

Logistic regression analysis identified the GCS score and mechanism of injury as significant predictors of mortality. Patients with a moderate GCS score were 8.1 times more likely to die than those with a mild score [OR = 8.07; 95% CI (3.69, 17.69); *P* < 0.001], while those with a severe GCS score were 61.2 times more likely to die [OR = 61.18; 95% CI (27.10, 138.13); *P* < 0.001]. Patients whose injuries were caused by falls were 3.4 times more likely to die compared to those injured in RTAs [OR = 3.40; 95% CI (1.37, 8.45); *P* < 0.008; Table 6].

**Table 6:** Factors influencing mortality risk: multivariate analysis of odds ratios

Predictors of mortality	OR	95 % CI	*P*-value
GCS score categorization			
Mild	REF		
Moderate	8.074	[3.686, 17.687]	**<0.001**
Severe	61.177	[27.095, 138.13]	**<0.001**
Mechanism of injury			
Road traffic accident	REF		
Assault	1.554	[0.806, 2.997]	0.188
Fall	3.398	[1.367, 8.451]	**0.008**
Other	3.115	[0.842, 11.527]	0.089
Work-related injury	1.412	[0.364, 5.474]	0.618
Alcohol use			
Yes	REF		
No	0.936	[0.451, 1.944]	0.859
Unknown	0.455	[0.183, 1.131]	0.09
Amnesia			
Yes	REF		
No	1.193	[0.3, 4.742]	0.802
Unknown	0.903	[0.217, 3.762]	0.889

## Discussion

This retrospective cohort study provides valuable insights into the epidemiology, severity, and short-term outcomes of TBI at a regional referral hospital in western Kenya. The demographic findings align with other studies in sub-Saharan Africa, showing a high incidence among young adult males^[3,4,8,9]^. This highlights the social and economic burden of TBI on the most productive demographic group in the region^[10]^.

### Injury mechanisms and prevalence of assault

While RTAs were the most common cause of TBI (61%), our finding that assault accounts for 27% of cases is particularly notable. This figure is higher than the global average (approximately 15%) and the national average (23.9%)^[3,11]^. The high prevalence of assault-related TBI suggests a significant contribution from interpersonal violence, a dimension of injury that is often under-discussed in the context of TBI in Kenya. This finding, associated with alcohol use, underscores the need for public health interventions that address social determinants of injury beyond road safety alone.

### Challenges in clinical management

The study’s findings on the low rates of imaging and neurosurgical intervention are concerning and reflect systemic challenges in low-resource settings. With only 34.2% of patients undergoing imaging, the true extent of intracranial injuries may be underestimated. While CT scanning is the standard^[12]^, its limited availability and the high cost for uninsured patients^[13]^ – notably, 65.5% of our cohort lacked insurance – likely contribute to this low utilization. Furthermore, the neurosurgical intervention rate of 2.8% is a major point of concern. A study in Uganda shows that patients with expansive intracranial hematomas who are candidates for surgical evacuation show significantly worse outcomes when surgery is delayed^[14]^. The lack of an on-site neurosurgeon and a dedicated intensive care unit at the facility likely resulted in the low intervention rates. This falls short compared to what is possible with improved neurosurgical capacity and referral systems.

### Outcomes

Our study’s mortality rates of 11.7% is somewhat lower than the pooled mortality from LMIC settings (approximately 16.7%) reported in a recent meta-analysis of over 50 000 TBI patients^[15]^. At Muhimbili Orthopedic Institute in Tanzania, severe TBI patients had mortality of approximately 30.7% among those with moderate to severe injury^[16]^. In South Africa, approximately 65.9% survived at discharge, but many of the survivors had moderate-to-severe disability^[17]^. This still reflects the huge challenge of managing severe injuries, especially with limited resources.

As expected, GCS score was a strong predictor of mortality, with severe TBI patients being 61 times more likely to die than those with mild TBI. A surprising finding was the significantly higher mortality risk from falls compared to RTA (OR = 3.40). This may be attributed to several factors. For example, falls in this cohort could be from significant heights, or they may occur in an older population with comorbidities that increase the risk of poor outcomes. The retrospective data, however, do not allow for a definitive causal explanation.

### Limitations

This study has several important limitations. First, as a retrospective cohort study, it is susceptible to selection bias and missing data, which was particularly evident for variables like education and insurance status. Second, the reliance on short-term outcomes at discharge fails to capture the long-term cognitive and functional disabilities that are a hallmark of TBI. Our study does not also account for prehospital deaths, which are likely substantial and represent a critical, uncaptured burden of TBI. Finally, the findings are based on a single hospital’s data and may not be generalizable to the entire country. However, they provide a valuable benchmark for similar facilities in comparable low-resource settings.

## Conclusion

This study confirms that TBI represents a significant public health burden at a regional referral hospital in western Kenya, predominantly affecting young adult males. RTAs are the primary cause of injury, but the high prevalence of assault-related TBI (27%) highlights a critical and under-addressed dimension of injury in this setting.

The findings underscore major systemic challenges, including limited access to diagnostic imaging and specialized neurosurgical care. The low rate of medical insurance coverage and the absence of an on-site intensive care unit further compromise patient outcomes, as reflected in the high mortality rate for severe TBI cases and those injured in falls.

While limited by its retrospective design and the reliance on short-term outcomes, this study provides important baseline data from a low-resource setting. The results offer compelling evidence to inform targeted public health interventions – such as enhanced road safety campaigns and initiatives to address violence – and advocate for strengthening the trauma care system. Ultimately, improving TBI management in this region will require a coordinated effort to address both injury prevention and health care system capacity.

## Data Availability

Datasets available upon reasonable request.

## Research registration unique identifying number (UIN)

This research was registered with the National Commission for Science, Technology and Innovation (NACOSTI), Kenya.
